# Risk factors for postoperative meningitis after microsurgery for vestibular schwannoma

**DOI:** 10.1371/journal.pone.0217253

**Published:** 2019-07-05

**Authors:** Bowen Huang, Yanming Ren, Chenghong Wang, Zhigang Lan, Xuhui Hui, Wenke Liu, Yuekang Zhang

**Affiliations:** Department of Neurosurgery, West China Hospital, Sichuan University, Chengdu, Sichuan, PR China; George Washington University, UNITED STATES

## Abstract

**Objective:**

Meningitis after microsurgery for vestibular schwannoma (VS) is a severe complication that results in high morbidity. However, few studies have focused on meningitis after VS surgery. The purpose of this study was to identify the risk factors for meningitis after VS surgery.

**Methods:**

We performed a retrospective analysis of all VS patients who underwent microsurgery and survived for at least 7 days after surgery, between 1 June 2015 and 30 November 2018 at West China Hospital of Sichuan University. Univariate and multivariate analyses were performed to identify the risk factors for postoperative meningitis (POM).

**Results:**

We enrolled 410 patients, 27 of whom had POM. Through univariate analysis, the factors of hydrocephalus (p = 0.018), Koos grade IV (p = 0.04), operative duration > 3 hours (p = 0.03) and intraoperative bleeding volume ≥400 ml (p = 0. 02) were significantly correlated with POM. The multivariate analysis showed that Koos grade IV (p = 0.04; OR = 3.19; 95% CI 1.032–3.190), operation duration > 3 hours (p = 0.03; OR = 7.927; 95% CI 1.043–60.265), and intraoperative bleeding volume ≥ 400 ml (p = 0.02; OR = 2.551; 95% CI 1.112–5.850) were the independent influencing factors of POM.

**Conclusions:**

Koos grade IV, operation duration > 3 hours, and intraoperative blood loss ≥ 400 ml were identified as independent risk factors for POM after microsurgery for VS. POM also caused a prolonged hospital stay.

## Introduction

Vestibular schwannoma (VS), also referred to as acoustic neuroma (AN), is a histopathologically benign tumor arising from Schwann cells surrounding the vestibular nerve[1]. The incidence of VS is estimated to be 1.9 per 100,000 per year[2]. Microsurgical resection is typically the gold standard of treatment for symptomatic, relatively young patients[3]. With the rapid development of minimally invasive neurosurgical technology and electrophysiological monitoring, the surgical mortality has significantly decreased[4]. However, the frequency of postoperative complications remains high. Meningitis is the main fatal complication after craniocerebral surgery. In addition, the data show that the incidence of postoperative meningitis (POM) following vestibular schwannoma surgery is approximately 5.5%-9.85%[4–8]. In the event of POM, the mortality can be as high as 50%[9]. However, related research on the clinical risk factors of meningitis after acoustic neuroma surgery is limited.

Therefore, we retrospectively analyzed the risk factors for meningitis following microsurgery for VS and the frequency of this complication. The results may be able to identify the factors that significantly affect POM and provide evidence for the prevention and early clinical treatment of POM.

## Materials and methods

### Patients

This study retrospectively enrolled 410 patients from the Department of Neurosurgery in West China Hospital of Sichuan University who underwent microsurgery for VS and survived at least 7 days after surgery between 1 June 2015 and 30 November 2018. We conducted this study from January to February 2019. The diagnosis was based on Magnetic resonance imaging (MRI) and pathology. MRI showed a mass arising from the vestibular nerve, and pathology showed schwannoma. This study was approved by the West China Hospital Ethics Committee. Written informed consent was exempt for the present study as a retrospective clinical study.

### Data collection

The basic patient information was collected, which included age, sex, BMI, signs and symptoms, presence/absence of diabetes mellitus, preoperative white blood cell count and hemoglobin concentration, presence/absence of hydrocephalus (assessed by MRI), side and size of the tumor, history of treatment of microsurgery and/or stereotactic radiosurgery for VS, duration of preoperative and postoperative hospitalization, surgery duration, bleeding amount during the operation, invasive operation, subcutaneous drainage and lumbar drainage as well as cerebrospinal fluid test results of patients who had cerebrospinal fluid drainage. The authors had access to information that could identify individual participants.

### Koos grade of VS

Koos grade was utilized for the classification of VS, according to magnetic resonance imaging findings and tumor size. VS was categorized into grades I, II, III and VI.

### Surgical technique

The surgical procedures were all performed by the senior neurosurgeon (YueKang Z). A retrosigmoid approach was used for all patients. The postauricular carbuncle was incised, the internal auditory canal was resected, and the tumor was totally or subtotally removed under a microscope. When electrophysiological monitoring was performed during the operation, precautions were taken to protect the trigeminal nerve, the facial nerve, the posterior cranial nerve and the brain stem. During the operation, prophylactic antibiotics and glucocorticoids were delivered, and glucocorticoids were given continuously until the third day after the operation.

### Definition of meningitis

Patients were required to meet at least 1 of the following criteria for the diagnosis of meningitis. 1. The patient had organisms cultured from cerebrospinal fluid (CSF). 2. The patient had at least 1 of the following signs or symptoms with no other recognized cause: fever (>38°C), headache, stiff neck, meningeal signs, cranial nerve signs, or irritability and at least 1 of the following criteria: a. increased white cells, elevated protein, and/or decreased glucose in CSF b. organisms seen on Gram’s stain of CSF c. organisms cultured from blood d. positive antigen test of CSF, blood, or urine e. diagnostic single antibody titer (IgM) or 4-fold increase in paired sera (IgG) for pathogens[10]. These data were obtained from cerebrospinal fluid samples collected by lumbar puncture or lumbar cistern drainage.

### Statistical analysis

All data were analyzed using SPSS software version 25.0 (IBM Corp., Armonk, New York, USA). Continuous variables are described by the median (range) and classified variables are described by percentages. The chi-squared test and Fisher’s exact probability test were employed to complete the univariate analysis of patients with and without meningitis. The significant factors (P <0.05) were entered into a multivariate logistic regression analysis to determine adjusted ORs. The relationship between POM and postoperative hospital stay was analyzed through the Wilcoxon-Mann-Whitney test. During this analysis process, all statistical tests were 2-sided; if P <0.05, the data were considered statistically significant.

## Results

### Baseline characteristics

In this study, 413 patients were enrolled, but three patients who died on postoperative day 1 were excluded. Some basic patient information is listed in Table 1. The mean age of the patients, including 172 men and 238 women, was 50 years old (range, 15–79 years). Their average BMI index was 23 kg/m^2^ (range, 16–38.6 kg/m^2^). According to the Koos grade, 8% of vestibular schwannomas are classified as the second level (II); 33.4% are classified as the third level (III); and 58.5% are classified as the fourth level (IV). The results show that 27 patients (6.6%) had POM, but no one died from the related meningitis.

**Table 1:** Patient characteristics and details of VS (vestibular schwannoma).

Variables	n (%) or median (range)
Age	50 (15–79)
Sex	
Male	172 (42%)
Female	238 (58%)
BMI	23 (16–38.6)
Side of VS	
Left	198 (48.3%)
Right	212 (51.7%)
Koos grade of VS	
I	0 (0%)
II	33 (8.0%)
III	137 (33.4%)
IV	240 (58.5%)

### Risk factors for POM

Through the univariate analysis (Table 2), hydrocephalus (p = 0.018), Koos grade IV (p = 0.04), operative duration > 3 hours (p = 0.03) and intraoperative bleeding volume ≥ 400 ml (p = 0. 02) were significantly correlated to POM. The multivariate analysis (Table 3) showed that Koos grade IV (p = 0.04; OR 3.19; 95% CI 1.032–3.190), operation duration > 3 hours (p = 0.03; OR 7.927; 95% CI 1.043–60.265), and intraoperative bleeding volume ≥ 400 ml (p = 0.02; OR 2.551; 95% CI 1.112–5.850) were the independent influencing factors of POM.

**Table 2:** Univariate analysis of the association between each factor and postoperative meningitis.

Variables	Postoperative meningitis		p value
	Yes (n = 27)	No (n = 383)	
Age			0.064^a^
>47	12(4.8%)	239(95.2%)	
≤47	15(9.4%)	144(90.6%)	
Sex			0.786^a^
Male	12(7.0%)	160(93.0%)	
Female	15(6.3%)	223(93.7%)	
BMI			0.128^a^
>23	8(4.5%)	171(95.5%)	
≤23	19(8.2%)	212(91.8%)	
Diabetes mellitus			0.057^b^
Present	3(21.4%)	11(78.6%)	
Absent	24(6.1%)	372(93.9%)	
Hydrocephalus			0.018^a^
Present	11(12.0%)	81(88.0%)	
Absent	16(5.0%)	302(95.0%)	
Side of tumor			0.435^a^
Left	15(7.6%)	183(92.4%)	
Right	12(5.7%)	200(94.3%)	
Previous stereotactic radiosurgery on the same side			1.000^b^
Yes	1(3.8%)	25(96.2%)	
No	26(6.8%)	358(93.2%)	
Previous microsurgery on the same side			0.657^b^
Yes	2(8.7%)	21(91.3%)	
No	25(6.7%)	362(93.3%)	
Preoperative hospitalization (days)			0.869^a^
>5	12(6.8%)	164(93.2%)	
≤5	15(6.4%)	219(93.6%)	
Koos grade of VS			0.004^b^
I-III	4(2.4%)	166(97.6%)	
IV	23(9.6%)	217(90.4%)	
Surgery duration(hours)			0.003^b^
>3	26(8.8%)	270(91.2%)	
≤3	1(0.9%)	113(99.1%)	
Bleeding amount of the operation (ml)			0.001^a^
≥400	12(15%)	68(85%)	
<400	15(4.5%)	315(95.5%)	
Subcutaneous drainage			0.053^a^
Present	13(10.1%)	116(89.9%)	
Absent	14(5%)	267(95%)	
Lumbar drainage			0.053^a^
Present	8(11.9%)	59(88.1%)	
Absent	19(5.5%)	324(94.5%)	
Preoperative white blood cell count (10^9^/L)			0.203^a^
>6	14(8.5%)	151(91.5%)	
≤6	13(5.3%)	232(94.7%)	
Preoperative hemoglobin concentration (g/L)			0.286^b^
>120	25(7.4%)	315(92.6%)	
≤120	2(2.9%)	68(97.1%)	

^a^ Chi-square test.

^b^ Fisher’s exact test.

**Table 3:** Multivariate analysis of factors associated with POM.

Variable	Odds ratio (95% CI)	p value
Hydrocephalus (present)	1.525(0.648–3.589)	0.333
Koos grade of VS (IV)	3.190(1.032–9.861)	0.044
Surgery duration (>3 hours)	7.927(1.043–60.265)	0.045
Bleeding volume during operation (≥400 ml)	2.551(1.112–5.850)	0.027

### Association between POM and length of postoperative hospitalization

The average length of hospital stay for patients with meningitis after surgery was 16.26 days, with a median of 13 days. The average length of hospital stay for patients without meningitis after surgery was 7.28 days, with a median of 6 days. There was a significant difference in postoperative hospital stay between the two groups of patients (p < 0.001).

### Pathogens of POM

In this study, the CSF of the 27 POM patients was cultured for bacteria and fungi. The culture time was three days. However, all the culture results were negative.

## Discussion

Meningitis is divided into aseptic meningitis and bacterial meningitis. According to a previous study, the positive rate of CSF culture is approximately 33%[11, 12]. However, in these meningitis patients, no bacteria were cultured in the CSF cultures, which may be due to the intraoperative prophylactic administration of an antibiotic and the prophylactic administration of antibiotics in the case of symptoms or signs of postoperative infection. The positive rate of CSF or blood culture decreases significantly if antibiotics are used for more than 24 hours before diagnosis[13]. In another study, the positive rate of CSF culture in their meningitis patients was also low, at 3.48% [4]. The culturing results may be negative, but many cases of culture-negative (aseptic) meningitis are bacterial meningitis[14]. In the future, we can try to improve the positive rate of CSF culture by some more sensitive methods, such as polymerase chain reaction techniques[14]. In this study, the incidence of meningitis after microsurgery of vestibular schwannoma was less than 6.6%, which is lower than the results reported by Huang Xiang. In their research, the incidence of meningitis after microsurgery of vestibular schwannoma is 9.85% [4]. What makes the results of the two studies inconsistent with each are the following observations. First, 8% of our patients had a tumor with a volume smaller than 30 x 20 mm. Second, we routinely used glucocorticoids intraoperatively and postoperatively to reduce cerebral edema and to suppress inflammatory responses. Through our data analysis, Koos grade, the duration of surgery and intraoperative blood loss were observed to be significant factors in the development of meningitis after microsurgery for vestibular schwannoma. In addition, POM significantly prolongs the length of hospital stay.

The current research shows that the size of acoustic neuroma can significantly affect the preservation of postoperative facial nerve function[15], cerebrospinal fluid leakage[16] and postoperative pneumonia[17]. However, no literature indicates that the size of acoustic neurons is related to POM. For the first time, our research points out that the size of the tumor significantly increased the risk of POM in patients with vestibular schwannoma. However, the specific mechanism correlating tumor size and POM remains unclear. Rahimpour, S. et al.[18] concluded that the contamination of CSF with bone dust related to drilling can lead to meningitis. During the resection of large VS, a large amount of bone chips will be produced by grinding away the internal auditory canal.

In addition, our study found that the duration of surgery significantly affected the occurrence of POM, which is consistent with the results of previous studies. Patir et al.[19] discovered that a surgical duration of more than 4 hours was significantly associated with higher postoperative infection rates. The mechanism may be that with prolonged operation time, the chance of external bacteria entering the cranium is increased, and the immune function of patients was inhibited under anesthesia. Dang, Y et al.[20] claimed that anesthesia affected the number and activity of immune cells and the secretion of cytokines. Meninges and soft tissues are retracted for surgical exposure, resulting in reduced perfusion and time-dependent reduced local immunological defenses[21]. With a prolonged operation time, the fatigue of the surgeon increases, which can easily lead to pollution of the operation area.

Intraoperative hemorrhage is also an independent risk factor for meningitis in patients with vestibular schwannoma after microsurgery. Chen, C et al.[22] and Chen, S et al.[23] studied the risk factors of meningitis after microsurgery but did not consider intraoperative hemorrhage as a risk factor. However, we found that an intraoperative bleeding volume > 400 ml increased the risk of postoperative meningitis by 2.551 times. The possible reason for this result entails further study, Naidech, A. M. et al.[24] believed that meningitis after operation was caused by subarachnoid congestion. Furthermore, blood easily accumulates in the cerebellopontine region after vestibular schwannoma microsurgery, leading to meningitis. At the same time, after massive intraoperative blood loss, allogeneic transfusion is often performed, but transfusion of allogeneic blood will complicate immune suppression [25]. The ability to fight infection is reduced in patients with decreased immunity, and meningitis is prone to occur. When patients have the above risk factors, once they have symptoms of infection, doctors should strongly suspect the occurrence of meningitis and incorporate the timely use of effective antibiotics to control the infection.

Once meningitis occurs after surgery, many studies report that the hospital stay of patients with acoustic neuroma after microsurgery will be significantly prolonged[26], which is consistent with the results of this study. In addition, medical expenses of patients are increased, the time to return to work is prolonged, and the burden on society is increased.

To prevent the complication of meningitis after operation for acoustic neuroma, the first step is to make an early diagnosis when the acoustic neuroma is still relatively small. Larger tumors are associated with more severe symptoms and surgical complications[27]. Improving surgeon proficiency and strengthening communication and logistics among surgical professionals can reduce the operation time. Golebiowski, A. et al.[21] confirmed that the duration of surgery may be shorter in older patients, so when younger patients are undergoing surgery, we should pay more attention to controlling the operation time. Intraoperative hemostasis is exact, and the traction of brain tissue should be reduced. Some studies have noted that administration of tranexamic acid(TXA) significantly reduced blood loss in patients undergoing elective craniotomy for the excision of intracranial meningioma[28]. At the same time, a new research pointed out that TXA administration significantly reduced postoperative infection rates, This effect was independent of the effect of TXA at reducing blood loss[29]. Ishihara, H. et al.[30] pointed out that preoperative embolization of meningioma can reduce intraoperative blood loss and surgery time. TXA administration and preoperative embolization may be used in microsurgery for VS in the future to shorten the operation time, reduce intraoperative bleeding and reduce the incidence of POM.

The disadvantage of this study is that it is a single-center study. There are admission deviations in our sample. In addition, surgical instruments and methods vary among hospitals, so our findings need to be verified in other hospitals. The average hospitalization time of our patients was only 7 days. Thus, patients suffering from meningitis after discharge were omitted from the statistics. Our study did not have any patients with positive cultures for bacteria and therefore failed to analyze the distribution of meningitis bacteria. Future studies with a larger sample and spanning multiple centers, as well as more rational prospective studies, are needed to analyze the bacterial species of meningitis to facilitate better treatment.

## Conclusion

In this study, the probability of meningitis after acoustic neuroma microsurgery was 6.6%. The risk factors of meningitis after microsurgery are Koos grade IV, operation duration >3 hours, and intraoperative bleeding volume ≥ 400 ml. In addition, POM has a significant association with an increase in hospitalization days after operation. Therefore, to prevent POM, TXA administration and preoperative embolization may be effective methods in the future.
