# Correction: Risk factors for postoperative meningitis after microsurgery for vestibular schwannoma

**DOI:** 10.1371/journal.pone.0226896

**Published:** 2019-12-17

**Authors:** Bowen Huang, Yanming Ren, Chenghong Wang, Zhigang Lan, Xuhui Hui, Wenke Liu, Yuekang Zhang

In the Funding section, the grant number from the funder Program of Sichuan Science and Technology Department is listed incorrectly. The correct grant number is: 2018SZ0143.
